# Th22 Cell Is a Gradually Proved Potential Biomarker for Acute Coronary Syndrome

**DOI:** 10.1155/2014/813926

**Published:** 2014-05-08

**Authors:** Ying Huang, Tao Xu, Jun Li

**Affiliations:** ^1^School of Pharmacy, Anhui Key Laboratory of Bioactivity of Natural Products, Anhui Medical University, Anhui, Hefei 230032, China; ^2^The Key Laboratory of Anti-Inflammatory and Immune, Anhui Medical University, Ministry of Education, Hefei 230032, China; ^3^Department of Cardiology, the First Affiliated Hospital of Anhui Medical University, Hefei 230032, China

Th22 cell, a novel human CD4^+^ T helper subset, has already been reported to play a vital role in the process of chronic inflammatory diseases such as rheumatoid arthritis (RA) and atherosclerosis (AS) [1, 2]. Interestingly, Th22 cells were also involved in acute coronary syndrome (ACS). Lin et al. found that circulating frequencies of Th22 cells and their effector cytokine IL-22 levels were manifestly increased in patients with acute myocardial infarction (AMI), unstable angina pectoris (UAP), and stable angina pectoris (SAP) compared with patients without coronary artery disease (CAD), indicating that plasma of Th22 cells played key roles at the early stage of ACS [3]. It was well accepted that CD4^+^ T helper cells were intimately associated with the process of ACS [4]; of note, Lin et al. firstly revealed the relationship between Th22 cells and ACS, providing a new and valuable evidence about the role of Th22 cells in ACS [1].

Encouragingly, similar finding was evident by another study which demonstrated that the circulating Th22 cells were remarkably higher in AMI and UAP patients than those in SAP patients and healthy people. Additionally, there was a positive correlation between the number of Th22 cells and IL-22 levels in AMI and UAP patients [5]. Moreover, study also showed that Th22 cells played pivotal roles in coronary plaque rupture or plaque erosion, since IL-22 was detected in the carotid plaque [6].

Taken together, these findings suggest that frequencies of Th22 cells are greatly increased in patients with ACS, indicating that the potential role of Th22 cells in the progression of ACS. Although large samples of clinical research are needed to further prove Th22 cell as a useful biomarker for ACS, the available evidences explore the close relationship between Th22 cells and the initiation of ACS, presenting a newly diagnostic potential for Th22 at onset of ACS.
